# Eosinophil microRNAs Play a Regulatory Role in Allergic Diseases Included in the Atopic March

**DOI:** 10.3390/ijms21239011

**Published:** 2020-11-27

**Authors:** Émile Bélanger, Anne-Marie Madore, Anne-Marie Boucher-Lafleur, Marie-Michelle Simon, Tony Kwan, Tomi Pastinen, Catherine Laprise

**Affiliations:** 1 Département des Sciences Fondamentales, Université du Québec à Chicoutimi, Saguenay, QC G7H 2B1, Canada; emile.belanger1@uqac.ca (É.B.); anne-marie_madore@uqac.ca (A.-M.M.); anne-marie1_boucher-lafleur@uqac.ca (A.-M.B.-L.); 2 Centre Intersectoriel en Santé Durable, Université du Québec à Chicoutimi, Saguenay, QC G7H 2B1, Canada; 3 Department of Human Genetics, McGill University, Montreal, QC H3A 0C7, Canada; marie-michelle.simon@mcgill.ca (M.-M.S.); tony.kwan@mcgill.ca (T.K.); tpastinen@cmh.edu (T.P.); 4 McGill University and Génome Québec Innovation Center, Montreal, QC H3A 0G1, Canada; 5 Center for Pediatric Genomic Medicine, Kansas City, MO 64108, USA

**Keywords:** microRNAs, miRNAs, eosinophils, atopic march, asthma, allergy, gene expression, sequencing

## Abstract

(1) Background: The atopic march is defined by the increased prevalence of allergic diseases after atopic dermatitis onset. In fact, atopic dermatitis is believed to play an important role in allergen sensitization via the damaged skin barrier, leading to allergic diseases such as allergic asthma and allergic rhinitis. The eosinophil, a pro-inflammatory cell that contributes to epithelial damage, is one of the various cells recruited in the inflammatory reactions characterizing these diseases. Few studies were conducted on the transcriptome of this cell type and even less on their specific microRNA (miRNA) profile, which could modulate pathogenesis of allergic diseases and clinical manifestations post-transcriptionally. Actually, their implication in allergic diseases is not fully understood, but they are believed to play a role in inflammation-related patterns and epithelial cell proliferation. (2) Methods: Next-generation sequencing was performed on RNA samples from eosinophils of individuals with atopic dermatitis, atopy, allergic rhinitis and asthma to obtain differential counts of primary miRNA (pri-miRNA); these were also analyzed for asthma-related phenotypes such as forced expiratory volume in one second (FEV_1_), immunoglobulin E (IgE) and provocative concentration of methacholine inducing a 20% fall in forced expiratory volume in 1 s (PC_20_) levels, as well as FEV_1_ to forced vital capacity (FEV_1_/FVC) ratio. (3) Results: Eighteen miRNAs from eosinophils were identified to be significantly different between affected individuals and unaffected ones. Based on counts from these miRNAs, individuals were then clustered into groups using Ward’s method on Euclidian distances. Groups were found to be explained by asthma diagnosis, familial history of respiratory diseases and allergic rhinitis as well as neutrophil counts. (4) Conclusions: The 18 differential miRNA counts for the studying phenotypes allow a better understanding of the epigenetic mechanisms underlying the development of the allergic diseases included in the atopic march.

## 1. Introduction

Allergic diseases (which include atopic dermatitis, food allergy, allergic asthma, and allergic rhinitis) are noncommunicable diseases that have seen the greatest increase in prevalence in the last decades, a phenomenon described as the “allergy epidemic” [1]. In the past ten years, allergy prevalence and hospitalization rates for severe allergic reactions has tripled [2] and our understanding of the root causes of allergic diseases has seen a complete paradigm shift [3]. In fact, the atopic march is defined by an increased prevalence of allergic diseases in individuals with atopic dermatitis. The latter, long considered only an epiphenomenon of allergies, has now clearly been shown to play a central role in their development. The inflamed and disrupted skin barrier of infants with atopic dermatitis allows the penetration of food and environmental allergenic proteins, leading to sensitization, the first step in becoming allergic. This explains why children with atopic dermatitis are at a high risk of developing food allergy (35% prevalence), allergic asthma (50%), and allergic rhinitis (75%) later in life [4]. Not only is the incidence of these diseases increased with atopic dermatitis, but they are also much more severe when compared to children without a history of atopic dermatitis [4].

Allergic asthma, which can happen at the final stages of the atopic march, is a chronic inflammatory disease characterized by symptoms of wheezing, shortness of breath, chest tightness, cough and variable expiratory airflow limitation [5]. Asthma is often associated with airway hyperresponsiveness and chronic inflammation in reaction to various stimuli including allergens, infections and air pollutants. Causes of asthma are multiple; they include environmental and genetic factors [6]. Indeed, familial history of asthma was found to be an important risk factor for the development of the disease, which is characterized by a calculated heritability of 55 to 90% [7]. However, heritability only partially explains the disease prevalence in the atopic march process. Another part of this prevalence could also be explained by epigenetic mechanisms such as DNA methylation, histone modification and non-coding RNAs. [8] More studies need to be conducted to better understand the influence of these mechanisms on diseases of the atopic march, especially concerning miRNA profiles.

miRNAs are small non-coding RNAs with 19–25 nucleotides that regulate gene expression post-transcriptionally. They silence complementary genes by targeting the 3′ untranslated region of mRNA, repressing their expression [9]. Their maturation includes primary (pri-miRNA, up to 1000 nucleotides long) and precursor (pre-miRNA, 60–120 nucleotides long) steps [10]. miRNAs act on various pro-inflammatory mechanisms in asthma and on smooth muscle cell proliferation, promoting airway hyperresponsiveness and playing an essential role in its pathogenesis [11]. Some miRNAs were also found to influence both the differentiation of T helper cells [12] and the innate immune response in keratinocytes of atopic dermatitis patients, [13] and to be differentially expressed in subjects with rhinitis and other allergic diseases [14,15].

Difference in miRNA profiles was also associated with other asthma-related phenotypes. Methacholine challenge (PC_20_), forced expiratory volume in one second (FEV_1_) and FEV_1_ to forced vital capacity (FEV_1_/FVC) ratio are all measures used as diagnostic tools or indicators of asthma severity [16,17]. Few studies have focused on miRNA expression predicting these phenotypes, but all three were previously associated with differential counts for certain miRNAs [18,19,20]. Finally, the differences in miRNA patterns that were observed for various diseases could lead to the identification of potential biomarkers for atopic dermatitis, asthma or other allergic diseases and even of potential therapeutic targets [8,11,21,22,23,24,25,26,27].

Moreover, few studies have demonstrated differential miRNA patterns in eosinophils, a type of pro-inflammatory cells whose proliferation in airways is characteristic of certain asthma phenotypes [28]. A study by Rodrigo-Muñoz et al. found that miRNAs in these cells that are peripheral can serve as potential diagnostic tools [29]. Another study by Allantaz et al. has shown a down-regulation of miR-155 in eosinophils that could be implicated in inflammatory processes [30]. Studying miRNA profiles from cell types well-known for their implication in allergic asthma is important considering the cell-type-specific characteristics of the transcriptome.

In this study, we sought to determine if some miRNAs were differentially expressed between individuals with allergic diseases included in the atopic march process and unaffected ones. We also wanted to see if they could be grouped based on their miRNA expression counts associated with diseases of the atopic march and phenotypes.

## 2. Results

**Figure 1:**
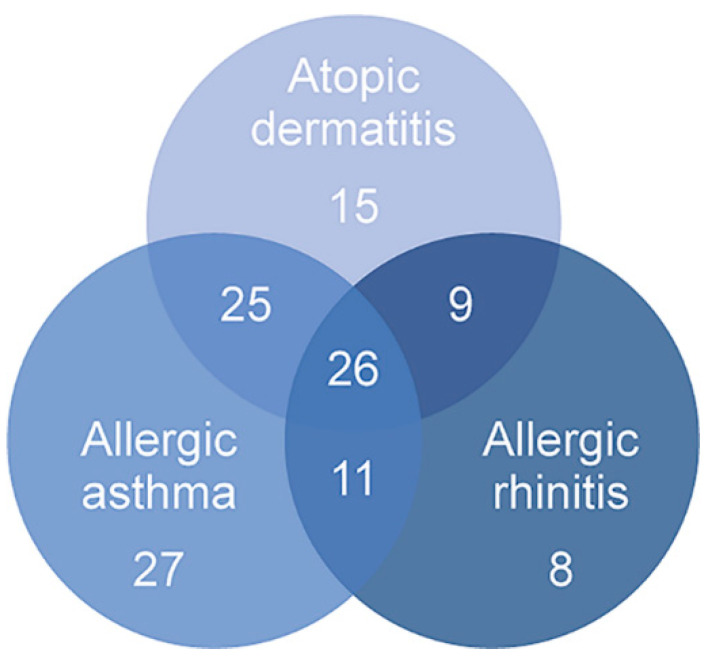
Number of individuals presenting overlapping conditions of the atopic march. Overall, 75 individuals presented atopic dermatitis, 89 had allergic asthma and 54 had allergic rhinitis. Several of these individuals had overlapping conditions, highlighting the increased risk of presenting an allergic disease as allergic asthma or allergic rhinitis if an individual has atopic dermatitis.

This study used pri-miRNA expression counts from 145 individuals of the Saguenay‒Lac-Saint-Jean (SLSJ) asthma familial cohort sampled for eosinophils in order to better understand underlying epigenetic mechanisms of diseases included in the atopic march process. The counts were analyzed for atopic dermatitis, atopy, allergic rhinitis, asthma, and asthma-related phenotypes (FEV_1_, IgE levels, PC_20_ and FEV_1_/FVC ratio). A clustering approach with significant miRNAs was then applied to identify potential explanations regarding the phenotypes for the differential counts of pri-miRNA between individuals. The phenotypic characteristics of the 145 individuals are accessible in Table 1 and number of individuals presenting overlapping conditions are represented in Figure 1.

### 2.1. Differential Counts of Pri-miRNA Associated with Allergic Diseases Included in the Atopic March

**Figure 2:**
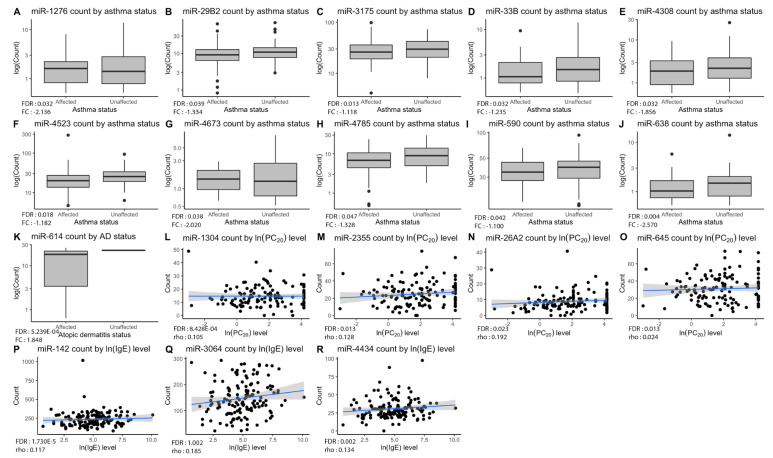
Significant pri-miRNA expression counts by phenotypes. Plots (**A**–**J**) show down-regulation of ten pri-miRNAs in asthmatic patients compared to non-asthmatics, while plot (**K**) shows up-regulation of miR-614 for individuals with atopic dermatitis in comparison with unaffected ones. As for plots (**L**–**O**), they display positive correlations between miRNAs and PC_20_ levels. Lastly, plots (**P**–**R**) show positive correlation between miRNAs and IgE levels. Overall *p*-value of the analysis is in lower-left corner of each graph with corresponding fold change or Spearman’s rho value.

The negative binomial regression model performed for each trait (atopic dermatitis, atopy, asthma, allergic rhinitis, FEV_1_, IgE levels, PC_20_ and FEV_1_/FVC ratio) allowed us to find 18 miRNAs with significant differences in expression counts for the eosinophil cell type (Table 2). One miRNA was found to be up-regulated in atopic dermatitis (fold change (FC) = 1.85), ten miRNAs were down-regulated in asthma (FC from −2.57 to −1.10), three miRNAs were positively correlated with IgE level (rho from 0.02 to 0.19) as well as four miRNAs with PC_20_ level (rho from 0.11 to 0.18). The distribution of counts for each significant miRNA in accordance with the testing phenotypes is represented in Figure 2.

### 2.2. Clustering of Significant miRNAs

**Figure 3:**
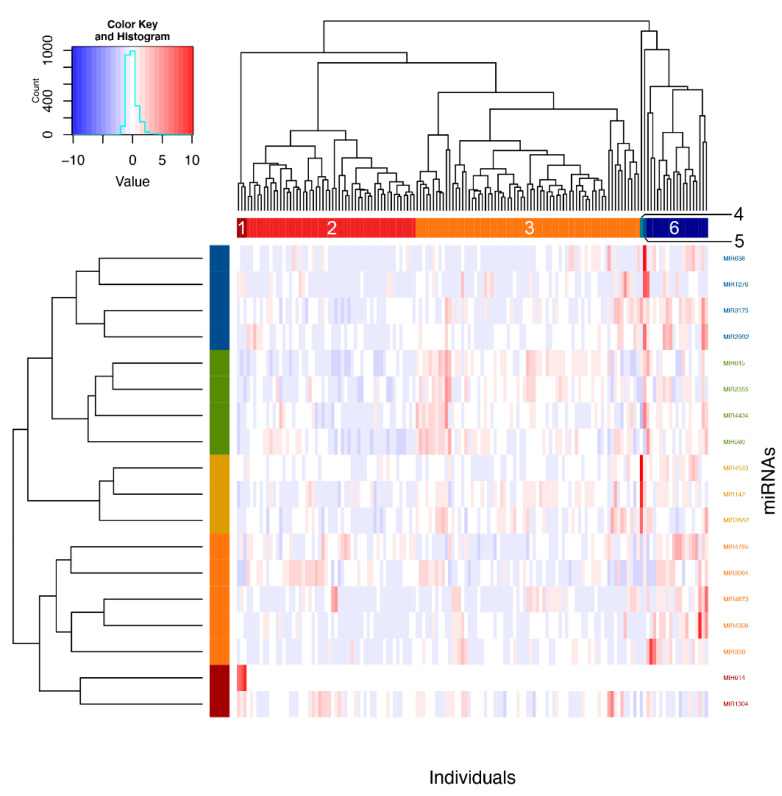
Clustering of individuals according to significant pri-miRNAs counts. Individuals were clustered according to expression counts from the 18 associated miRNAs using Ward’s method on Euclidian distances. Six clusters were then obtained by visual inspection of the dendrogram and confirmed with the NbClust package in R.

**Figure 4:**
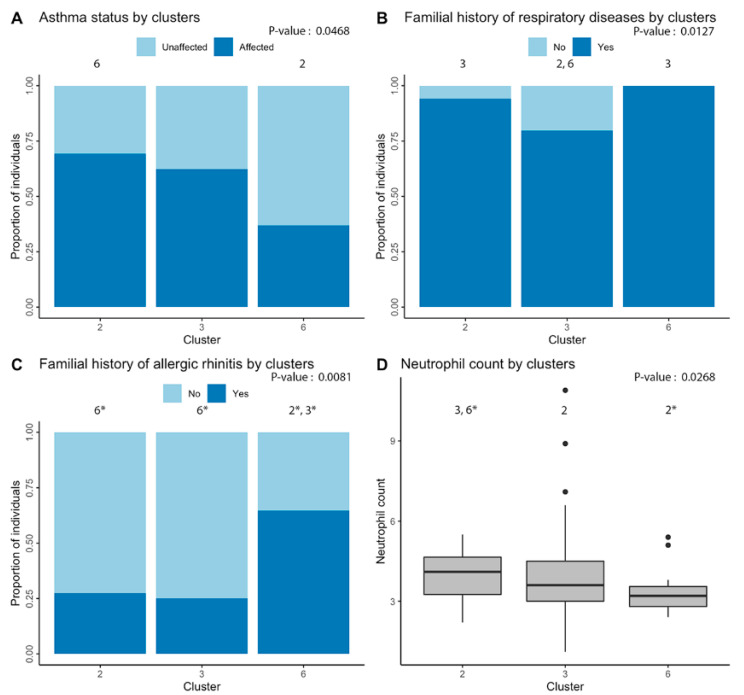
Phenotypic differences underlying clustering of individuals. Significant differences between individuals of the three main clusters (including more than three people) were found for: (**A**) asthma; (**B**) familial history of respiratory diseases; (**C**) familial history of allergic rhinitis; and (**D**) neutrophil cell count. Overall *p*-value of the analysis is in the upper-right corner of each graph and the number above each cluster indicates the other clusters that are significantly different in post-hoc analyses. An asterisk indicates significant difference after Bonferroni correction.

Using Ward’s method on Euclidian distances for the significant miRNAs, six clusters were obtained (Figure 3). Analyses were then performed to define which phenotypic elements characterize them. Clusters 1, 4 and 5 were ignored in analyses because they contained three individuals or less. As for clusters 2, 3 and 6, they included, respectively, 52, 69 and 19 individuals and were kept for subsequent steps. See Table S1 for a detailed view of all the phenotypes for comparing clusters. These were found to be explained by asthma phenotype, familial history of respiratory diseases and allergic rhinitis as well as neutrophil counts (Figure 4).

Moreover, looking at Figure 4 and according to post-hoc analyses, cluster 2 was associated with higher rates of diagnosis of asthma as compared with cluster 6 (*p*-value = 0.027). Cluster 3 was less related to familial history of respiratory diseases as clusters 2 (*p*-value = 0.033) and 6 (*p*-value = 0.034). Cluster 6 also had a stronger association with familial history of allergic rhinitis when compared with clusters 2 (*p*-value = 0.009) and 3 (*p*-value = 0.003). Finally, cluster 2 had a higher neutrophil count in comparison with clusters 3 (*p*-value = 0.035) and 6 (*p*-value = 0.005).

### 2.3. Identifiaction of Possible Gene Targets

**Figure 5:**
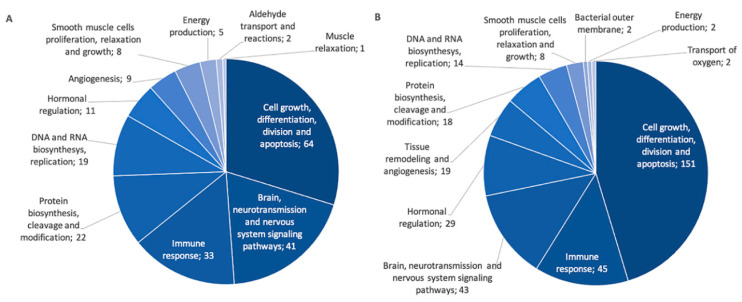
General categories of PANTHER pathways for gene targets identified in negative and positive correlations with associated miRNAs. The number of genes is indicated for each category. (**A**) Pathways for negatively correlated miRNA–gene target pairs. (**B**) Pathways for positively correlated miRNA–gene target pairs.

In order to better understand underlying mechanisms explaining associations observed between pri-miRNA expression and diseases of the atopic march or clinical measures, correlations were performed to identify possible gene targets. miRNA–gene target pairs with false discovery rate (FDR)-values inferior to 0.05 and absolute correlation coefficients >0.2 were deemed significant and further validated using miRTArBase v8.0 [31]. A total of 540 validated miRNA–gene target pairs were found using negative correlations and 663 using positive correlations. According to the inhibition function of miRNAs, results of negative correlations are usually prioritized. However, a recent study highlighted the mechanisms underlying positive correlations with gene targets, such as in feedback loops and co-transcription of intronic miRNAs and their host genes [32], motivating the presentation of positive correlations in this study. The top 25 best negative and positive correlations are displayed in Table S2. They were then classified into PANTHER biological pathways [33]. Among the gene targets identified, 215 from the negative correlations and 353 from the positive ones have been successfully classified by PANTHER. Biological pathways identified were grouped into more general categories shown in Figure 5 (see Table S3 for the list of PANTHER pathways in each general category).

Finally, gene targets found were compared to the genes already associated with diseases of the atopic march in the GWAS Catalogue (42 for negative correlations and 53 for positives ones) and the SLSJ asthma familial cohort (13 for the negative correlations and 22 for the positive ones; Table S4). Table 3 represents those gene targets that are included in PANTHER biological pathways. Negative correlations included eight gene targets associated in GWAS analyses and four were associated in the SLSJ cohort. Positive correlations included 19 gene targets associated in GWAS and nine were associated in the SLSJ cohort. Total resulting number of targets for positive and negative correlations before and after validation, as well as number of genes found in GWAS Catalogue and literature about the SLSJ asthma familial cohort are detailed in Table S4.

## 3. Discussion

This research used pri-miRNA counts obtained by next-generation sequencing of the whole eosinophil transcriptome, a cell known to play an inflammatory role in asthma [28], for finding differential expression of certain miRNAs between patients having allergic diseases often associated with the atopic march and controls in order to better understand epigenetic mechanisms underlying these phenomena. Specifically, it was previously noted that eosinophils can carry miRNAs via exosomes to other cells; they are becoming not only a pro-inflammatory cell type, but also a vector for miRNAs and a potential regulator of gene expression [34]. In this sense, extracting miRNAs from eosinophils allows a better understanding of their effect on diseases in the atopic march process as compared to those from whole blood. Overall, miRNAs extracted from eosinophils improve the knowledge of their possible roles in proliferation of the inflammatory cells and immune functions in these diseases [35,36].

The miRNA levels measured from eosinophil cells extracted as part of this study allowed us to identify 18 that were differentially expressed between individuals with allergic diseases and unaffected ones, as well as for IgE levels and PC_20_. Five of them were associated in the past with asthma and allergic diseases (miR-142 [37,38], -26a [39,40], -29b [41,42], -590 [29,43] and -638 [44]). In fact, the miRNA expression counts of these five miRNAs were all differentially regulated in the same direction as that is found in literature. Three were previously associated with other respiratory conditions including lung cancer (miR-1276 [45], -1304 [46] and -33b [47]), hypopharyngeal cancer (miR-1304 [48]) and cystic fibrosis (miR-1276 [49]). Finally, three were previously associated with diseases involving inflammatory components (miR-2355 [50], -3175 [51], -33b [52]). This study led to the identification of 13 miRNAs not previously associated with allergic diseases or asthma. The remaining five miRNAs were already found to have a differential expression in diseases of the atopic march, but this study allowed the confirmation that these were also differentially expressed in eosinophils. There are very few studies on miRNA expression in eosinophil samples for allergic diseases. A study by Rodrigo-Muñoz et al. found 21 miRNAs differentially expressed in eosinophils in asthma, from which miR-590 was also down-regulated [29]. No significant differences were observed for the 20 other miRNAs in our study. This could be explained by their smaller sample size (29 asthmatic and 10 healthy individuals), the fact that they did not study an allergic endotype of asthma as done in this project, and also because we measured differential pri-miRNA counts as compared to mature miRNAs in their study. However, measuring pri-miRNA counts allow a more direct understanding of miRNA transcription. Finally, even though no mature miRNA data were available, several pri-miRNAs were previously associated with mature miRNAs in allergic diseases included in the atopic march process. Moreover, the target analysis of the significant miRNAs associated in this study found *CITED2* as a target of miR-590, a gene regulating TGF-beta pathways acting on airway smooth muscle cell proliferation. In fact, eosinophils have previously been shown to enhance gene expression of TGF-beta1 and to increase airway smooth muscle cell proliferation [53]. In this sense, it is expected that this miRNA can be differentially regulated in both allergic and non-allergic endotypes of asthma. These findings point toward a regulatory role in these diseases. Finally, considering modest coefficients of correlations found between pri-miRNA counts and PC_20_ and IgE levels, we hypothesize that such correlations are more complex than mere direct interaction. In fact, Davis et al. have found that certain miRNAs could modulate PC_20_ by increasing airway smooth muscle cell diameter [18]. Therefore, more studies need to be done in order to better define links between these miRNAs and biological measures.

Actually, some well-known miRNAs in asthma and allergic diseases such as miR-221, -485-3p, -1248, -126, -146a/b, -28-5p, -181a, -133a or -10a [23] were not replicated in this study. Among these, four miRNAs were expressed in eosinophil samples, but their corresponding pri-miRNAs did not show significant differences according to the phenotype status. However, based on the cell-type specificity of transcriptome measurements, it was expected that eosinophils will have a distinct profile in comparison to whole blood. It was demonstrated in the past that whole blood miRNAs are derived from various exosomes specific to cell types; this means previously found differential miRNA counts associated with diseases in the atopic march process could be derived from other ones like lymphocytes, dendritic cells, platelets, mastocytes, epithelial cells, endothelial cells or neurons [54].

To further understand the links between pri-miRNA expression counts and phenotypic differences in asthma and allergic diseases, individuals were clustered into three distinct groups based on the pri-miRNA counts for each of the 18 previously associated miRNAs. These clusters featured significant differences, which is consistent with the proportion of subjects having asthma phenotype as well as familial history of respiratory diseases and allergic rhinitis, further confirming the family component in them [6]. Moreover, earlier studies have associated asthma prevalence with allergic rhinitis [55,56,57]; these findings tend to demonstrate a link between the two. However, more studies need to be done in regard to the similarities between the eosinophil miRNA patterns between asthma and allergic rhinitis to reach such a conclusion. A difference in counts of circulating neutrophils, a cell type characterizing an asthma endotype not typically linked with allergies [58], was also observed. With a higher proportion of individuals with familial history of allergic rhinitis and a smaller neutrophil count, cluster 6 shows a higher number of allergic individuals, even though its proportion of asthmatics is lower. However, it is important to note the smaller number of individuals included in this cluster (19 compared to 52 and 69). Interestingly, many miRNAs turned out to be differentially regulated in cluster 6, as compared to clusters 2 and 3. These miRNAs were previously found to play a role in diseases of the atopic march. It is the case for miR-142 [37,38], -26a [39,40], -29b [41,42], -590 [29,43] and -638 [44] which all had higher counts in cluster 6, in comparison with clusters 2 and 3. Similarly, both clusters 2 and 3 showed higher values for miR-1276 and -1304 whereas cluster 3 showed higher values for miR-33b and -2355, four miRNAs known for their implication in non-allergic respiratory diseases [45,46,47,48,49,50]. Furthermore, no association was made with eosinophil counts in the clustering approach. This could be explained by the fact that the eosinophil cell counts were similar between affected (by one or more allergic disease) and non-affected (by none of the allergic diseases tested) individuals. However, there is indeed a difference in eosinophil cell counts when we compare for a single disease such as asthma, with a mean of 0.29 × 10^9^/L eosinophils in individuals with asthma and 0.18 × 10^9^/L in unaffected ones. However, miRNAs associated in eosinophil samples were not necessarily linked with biological pathways involved in eosinophil recruitment or proliferation.

To further understand the implication of these miRNAs on diseases of the atopic march, an analysis to identify possible gene targets was performed. The 18 associated pri-miRNA expression counts were significantly correlated with several targets previously identified in diseases of the atopic march. Overall, 95 gene targets were already associated with diseases of the atopic march in the GWAS Catalogue and 35 with the SLSJ asthmatic cohort. Among gene targets that were linked with biological pathways, 42 were also associated with diseases of the atopic march, either in the GWAS Catalogue or in the SLSJ cohort. Those genes were classified into pathways including cell regulation, immune response, smooth muscle cell proliferation and angiogenesis, further confirming the importance of miRNAs in these diseases. However, further studies are needed to better understand the specific link between these 18 miRNAs and the different diseases of the atopic march.

This study sought to find a differential miRNA pattern in eosinophils from patients presenting with diseases in the atopic march process and unaffected individuals. Eighteen miRNAs turned out to be differentially expressed in eosinophil samples in case of either atopic dermatitis or asthmatic statuses than in unaffected individuals, or according to the PC_20_ or IgE levels. Moreover, the clustering approach used with the associated miRNAs revealed a link between these and fine phenotypic information defining the individuals included in this study and allowed identifying the miRNAs that are more likely to be involved specifically in the atopic component of the studied diseases among the 18 in association.

Overall, these miRNAs could help improve the knowledge of post-transcriptional regulation leading to allergic diseases included in the atopic march process. In fact, their differential regulation found in eosinophils help refine our understanding of miRNAs in these diseases and their important role in gene expression regulation. Finally, the presence of multiple atopic diseases in one patient may imply the relevance of miRNAs in the atopic march. However, birth cohorts and longitudinal studies will need to be performed to support this hypothesis. Despite this, it is interesting to note that a previous study including the SLSJ cohort and a birth cohort aiming at developing a polygenic risk score for moderate-to-severe atopic dermatitis, the sub-phenotype of atopic dermatitis associated with the highest risk to develop the atopic march, also showed good discriminative values for allergies, allergic asthma and allergic rhinitis in the SLSJ cohort [59].

## 4. Materials and Methods

### 4.1. SLSJ Asthma Cohort

The SLSJ asthma familial cohort comprises 1394 individuals distributed in 271 families from which 1214 subjects have genotypic information. Pulmonary health of each individual was evaluated according to the American Thoracic Society (ATS) Clinical Practice Guidelines using a standardized questionnaire and pulmonary function testing [60]. The subpopulation used for this study was selected using siblings with discordant asthma status and from trios of affected probands and discordant parents regarding asthma status. Using these criteria, 215 subjects were obtained to isolate their eosinophils for RNA sequencing. Asthma and atopy phenotypes were defined according to ATS standards [61]. Participants were considered as asthmatic if: (1) they had a reported history of asthma (validated by a physician), or (2) they presented asthma-related symptoms and positive PC_20_ (<8 mg/mL) at recruitment. Individuals were deemed atopic if they had at least one positive response on skin prick tests (wheal diameter ≥ 3 mm or larger than the wheal diameter elicited by the negative control (glycerin)) and have a physician diagnosis. Atopic dermatitis and allergic rhinitis were self-reported and were considered as positive if past or present occurrence of these diseases was reported. For children, cross validation was done using questionnaires filled out by their parents. Moreover, validation in medical records of these self-reported phenotypes were done for a subset of the SLSJ Cohort (*n* = 217), giving 89% concordance. Proportions of white blood cells were obtained using a Coulter LH 780 hematology analyzer (Beckman Coulter, Mississauga, ON, Canada) to estimate proportion of five different types of white blood cells: eosinophils, lymphocytes, neutrophils, monocytes and basophils. Respiratory measures such as PC_20_ and FEV_1_ were taken using a Morgan spirometer (Morgan Spiro 232, P.K. Morgan Ltd., Kent, UK) [62]. Complete descriptions of both recruitment and evaluation used for the SLSJ cohort can be found in Laprise et al. [62] All participants gave informed consent and the study was approved by the Centre intégré universitaire de santé et de services sociaux du Saguenay–Lac-Saint-Jean ethics committee (project #0002-001, 08-11-2005).

### 4.2. Isolation of RNA from Eosinophils and Sequencing

Complete description of the procedure for eosinophil isolation can be found in Madore et al. [63] Briefly, eosinophils were isolated by negative selection from 200 mL blood samples with anti-CD16, anti-CD3 and anti-CD19 MicroBeads (Miltenyi Biotec, Auburn, CA, USA) and a proportion of cells (2 × 10^6^) was used for total RNA extraction (molecules ≥ 200 nucleotides). This step was performed with the RNeasy Mini Kit following manufacturer’s instructions (Qiagen, Toronto, ON, Canada). RNA sequencing was carried out at the McGill University and Genome Québec Innovation Center using TruSeq Stranded Total RNA Sample Prep kit (Illumina, Vancouver, BC, Canada). Final libraries were quality controlled on a Bioanalyzer (Agilent Technologies, Mississauga, ON, Canada) and underwent 100 bp paired-end sequencing on the Illumina HiSeq2000 System (Illumina, Vancouver, BC, Canada). Generated raw reads were filtered for quality (phred33 ≥ 30) and length (*n* ≥ 32) as well as adapter sequences were removed using Trimmomatic v.0.32 as previously described [63]. Considering the length of the extracted RNAs (≥200 nucleotides), expression counts were available for pri-miRNAs. After quality control filtering, 441 pri-miRNA were available for analyses. From the 215 initial individuals, considering the large amount of blood necessary for RNA sequencing, quality filtering applied and the covariates availability for statistical analysis, pri-miRNA counts were accessible for 145 of them. These results reflect the transcription of miRNA sequences without necessarily quantifying the abundance of the mature miRNAs [10].

### 4.3. Statistical Methods

#### 4.3.1. Analyses of Pri-miRNA Expression Counts between Individuals

Pri-miRNA expression counts were compared with a negative binomial regression model built with pri-miRNA normalized expression counts and phenotypes using the MASS package in R. Studied phenotypes were atopic dermatitis (75 affected, 70 healthy), atopy (94 affected, 51 healthy), allergic rhinitis (54 affected, 91 healthy), asthma (89 affected, 56 healthy), FEV_1_, IgE levels, PC_20_ and FEV_1_/FVC ratio. Age, sex, smoking history, eosinophil proportion and surrogate variables were used as covariates. Proportions of eosinophils were calculated working with methylome data from the same eosinophil samples as well as the method by Houseman implemented in the R package RnBeads as described above (Table S5) [64,65]. Surrogate variables were evaluated using the R package sva in order to account for batch effects, relatedness between samples and other hidden confounders [66]. Normalization of miRNAs expression counts on library size was done with the DESeq2 package. Differences in these with a FDR-value <0.05 were deemed significant. FC were calculated using the mean ratios for affected and non-affected individuals in case of analyses taking into account the disease status whereas Spearman’s rank correlation was performed between pri-miRNA counts and continuous phenotypes. miRNAs with positive FC were considered up-regulated while those with negative FC were down-regulated in the affected group of each allergic disease analyzed.

#### 4.3.2. Clustering of miRNAs

Significant miRNAs were used to cluster individuals with Ward’s method on Euclidian distances in R, as described by O’Sullivan et al. [67] Groups were determined by visual inspection of the dendrogram and confirmed using the NbClust package in R. An explanation for these clusters was then searched comparing the phenotypic measures between groups with Fisher’s exact test for binary data, one-way ANOVA for continuous normally distributed variables and Kruskal–Wallis test for continuous non-normally distributed ones. Post-hoc analyses were performed afterward to find pairwise differences between clusters using Fisher’s exact test, Tukey’s test and Dunn’s test, respectively, followed by Bonferroni corrections. Clusters with three individuals or less were ignored from this testing (data available for 139 subjects on the 145 included in the pri-miRNA analysis).

#### 4.3.3. Identification of Possible Gene Targets for Associated Pri-miRNAs

Negative and positive correlations were applied between the 18 associated pri-miRNA and gene log-transformed expression count in order to find which genes are possible gene targets. Correlations that reached an FDR-value <0.05 and an absolute correlation coefficient > 0.2 were deemed significant. These miRNA–gene target pairs were then validated with miRTarBase v8.0 [31], a database that list gene targets of miRNAs that were observed using technical approaches (e.g., qRT-PCR, next-generation sequencing), in order to find which of the significant genes are more likely to be real targets. These targets were classified according to their PANTHER pathways [33]. Gene targets that were previously associated with diseases of the atopic march were identified using the GWAS Catalogue (https://www.ebi.ac.uk/gwas/) and studies from the SLSJ asthma familial cohort. The GWAS Catalogue list all human genome-wide associations for studies targeting 100,000 single nucleotide polymorphisms (SNPs) or more and with *p*-values <10 × 10^−5^.

## Figures and Tables

**Table 1:** Phenotypic characteristics of the individuals included in the eosinophil analysis of the miRNAs.

	Eosinophil Samples ^a^ (*n* = 145)	Affected ^b^ (*n* = 130)	Unaffected ^c^ (*n* = 15)
M:F ratio	1:1.04	1:1.09	1:0.67
Age, mean (range)	46 (18–81)	45 (18–81)	55 (22–72)
Age, median	47	60	44
Smoking status ^d^			
Non-smokers, n (%)	93 (64)	88 (68)	5 (33)
Ex-smokers, n (%)	29 (20)	22 (17)	7 (47)
Smokers, n (%)	22 (15)	19 (15)	3 (20)
PC_20_, mean mg/mL (SD) ^e^	1.76 (1.67)	1.5 (1.57)	3.75 (0.76)
IgE, mean µg/L (SD) ^f^	4.93 (1.67)	5.09 (1.67)	3.61 (0.97)
FEV_1_, mean% pred. (range) ^g^	94.72 (31–146)	94.25 (31–46)	98.6 (65–119)
FEV_1_/FVC ratio, mean (range) ^h^	79.09 (38–97)	94.25 (38–97)	82.43 (68–96)
White blood cell count ^i^			
Eosinophil, mean × 10^9^/L (%)	0.25 (3.76)	0.26 (3.96)	0.15 (2.18)
Lymphocyte, mean × 10^9^/L (%)	2.15 (32.12)	2.15 (32.05)	2.03 (31.43)
Monocyte, mean × 10^9^/L (%)	0.52 (7.89)	0.53 (7.88)	0.50 (8.01)
Neutrophil, mean × 10^9^/L (%)	3.80 (55.60)	3.82 (55.45)	3.72 (57.89)
Basophil, mean × 10^9^/L (%)	0.04 (0.78)	0.05 (0.77)	0.04 (0.89)
Asthma, n (%) ^j^	89 (61.81)		
Allergic rhinitis, n (%) ^k^	54 (37.50)		
Atopic dermatitis, n (%) ^l^	75 (52.08)		

^a^ Number of eosinophil samples for which the sequencing of whole transcriptome data as well as covariates for analyses was available. ^b^ Number of affected individuals. Total affected individuals are individuals with either atopic dermatitis, atopy, asthma or allergic rhinitis, or a combination of these diseases. In this sense, depending on the studied phenotype, the number of affected individuals will be lesser while the number of unaffected individuals will be higher. ^c^ Number of unaffected individuals. Unaffected individuals were individuals with no disease of the atopic march. ^d^ Ex-smokers are defined as individuals who stopped smoking for at least one year. Smoking status was not available for one individual. ^e^ The geometric mean provocative concentration of methacholine inducing a 20% fall in forced expiratory volume in 1 s (PC_20_), calculated from 130 individuals. ^f^ The geometric mean of immunoglobulin E (IgE) levels measured from 142 individuals. ^g^ The mean forced expiratory volume in 1 s (FEV_1_) as % of predicted value calculated from 138 individuals. ^h^ The mean FEV_1_ (L)/FVC (forced vital capacity; L) ratio calculated as % for 128 individuals. ^i^ Whole blood eosinophil, lymphocyte, monocyte, neutrophil and basophil cell counts obtained using a Coulter LH 780 hematology analyzer. ^j^ Present or past documented clinical history of asthma. Status is available for 145 individuals. ^k^ Personal history of allergic rhinitis symptoms available for the 145 individuals. ^l^ Personal history of atopic dermatitis available for the 145 individuals.

**Table 2:** Significant associations (FDR < 0.05) between pri-miRNAs expression counts and diseases included in the atopic march process or related phenotypes.

miRNA	Phenotype Observed	*p*-Value	FDR ^a^	FC or rho ^b^
miR-1276	Asthma	4.511 × 10^−4^	0.032	−2.136
miR-29B2	Asthma	7.260 × 10^−4^	0.040	−1.334
miR-3175	Asthma	5.920 × 10^−5^	0.013	−1.118
miR-33B	Asthma	4.971 × 10^−4^	0.032	−1.235
miR-4308	Asthma	5.039 × 10^−4^	0.032	−1.856
miR-4523	Asthma	1.195 × 10^−4^	0.018	−1.182
miR-4673	Asthma	2.164 × 10^−4^	0.024	−2.020
miR-4785	Asthma	0.001	0.047	−1.328
miR-590	Asthma	8.479 × 10^−4^	0.041	−1.100
miR-638	Asthma	1.010 × 10^−5^	0.004	−2.570
miR-614	Atopic dermatitis	1.190 × 10^−6^	5.239 × 10^−4^	1.847
miR-142	IgE	3.940 × 10^−8^	1.730 × 10^−5^	0.112
miR-3064	IgE	4.880 × 10^−5^	0.007	0.185
miR-4434	IgE	4.060 × 10^−5^	0.007	0.134
miR-1304	PC_20_	1.920 × 10^−6^	8.428 × 10^−4^	0.105
miR-2355	PC_20_	7.120 × 10^−5^	0.013	0.128
miR-26A2	PC_20_	2.054 × 10^−5^	0.023	0.192
miR-645	PC_20_	8.540 × 10^−5^	0.013	0.024

^a^ Significance level for the difference of pri-miRNA expression counts according to phenotypic traits corrected using a false discovery rate (FDR) method. ^b^ Fold change (FC) or Spearman’s rho calculated depending on the phenotypic data type. Fold change was calculated for asthma and atopic dermatitis phenotypes. miRNAs with a positive fold change were considered up-regulated in affected individuals while miRNAs with a negative fold change were down-regulated. Spearman’s rho was calculated for IgE and PC_20_ levels. Positive rho values indicate positive correlations between the pri-miRNA counts and the phenotype.

**Table 3:** miRNAs’ gene targets previously associated in GWAS as listed by the GWAS Catalogue or in the Saguenay‒Lac-Saint-Jean (SLSJ) cohort and classified by PANTHER pathways.

	Pathway	Nb of Gene Targets	GWAS Associated Genes ^a^	SLSJ Associated Genes ^a^
Negative correlation	Cell growth, division, differentiation and apoptosis	64	COL15A1, SOCS1, *BCL2L1*, *DUSP2*, *SSR3*	COL15A1, SOCS1
	Immune response	33	*FER*, *IL6*	
	Protein cleavage, biosynthesis and modification	21		*BACE2*
	DNA and RNA synthesis and replication	19		*MAT2A*
	Aldehyde transport and reactions	2	*DCAKD*	
Positive correlation	Cell growth, division, differentiation and apoptosis	158	*APC*, *CASP8*, *CRK*, *DUSP2*, *FOS*, *FRS2*, HSPA1B, *PRKCD*, *RTF1*, *SKI*, *TGFBR1*	HSPA1B, *HSPA6*, *YWHAZ*
	Brain, neurotransmission and nervous system	44	*ALDH1A2*, *CDC42*	*ARHGEF1*
	Immune response	38	*REL*, *TNFA1P3*	*CDNK1B*, *STAT3*
	Protein cleavage, biosynthesis and modification	35	*INO80*, *LRP3*	*MMP9*
	Hormonal regulation	29	*NAB2*, *POU2F1*	*LDB1*, *SP1*

^a^ Genes in blue are those that are associated with allergic diseases included in the atopic march according to the GWAS Catalogue and as well as in studies performed in the Saguenay‒Lac-Saint-Jean (SLSJ) asthma familial cohort.

## Supplementary Materials

Supplementary Materials can be found at https://www.mdpi.com/1422-0067/21/23/9011/s1.
